# Up in the Tree – The Overlooked Richness of Bryophytes and Lichens in Tree Crowns

**DOI:** 10.1371/journal.pone.0084913

**Published:** 2013-12-17

**Authors:** Steffen Boch, Jörg Müller, Daniel Prati, Stefan Blaser, Markus Fischer

**Affiliations:** 1 Institute of Plant Sciences and Botanical Garden, University of Bern, Bern, Switzerland; 2 Institute of Biochemistry and Biology, University of Potsdam, Potsdam, Germany; University Copenhagen, Denmark

## Abstract

Assessing diversity is among the major tasks in ecology and conservation science. In ecological and conservation studies, epiphytic cryptogams are usually sampled up to accessible heights in forests. Thus, their diversity, especially of canopy specialists, likely is underestimated. If the proportion of those species differs among forest types, plot-based diversity assessments are biased and may result in misleading conservation recommendations. We sampled bryophytes and lichens in 30 forest plots of 20 m × 20 m in three German regions, considering all substrates, and including epiphytic litter fall. First, the sampling of epiphytic species was restricted to the lower 2 m of trees and shrubs. Then, on one representative tree per plot, we additionally recorded epiphytic species in the crown, using tree climbing techniques. Per tree, on average 54% of lichen and 20% of bryophyte species were overlooked if the crown was not been included. After sampling all substrates per plot, including the bark of all shrubs and trees, still 38% of the lichen and 4% of the bryophyte species were overlooked if the tree crown of the sampled tree was not included. The number of overlooked lichen species varied strongly among regions. Furthermore, the number of overlooked bryophyte and lichen species per plot was higher in European beech than in coniferous stands and increased with increasing diameter at breast height of the sampled tree. Thus, our results indicate a bias of comparative studies which might have led to misleading conservation recommendations of plot-based diversity assessments.

## Introduction

Assessing diversity is a major task in ecology and conservation science. Understanding patterns of diversity and guiding conservation decision-making both rely on an unbiased assessment of diversity, for instance of species richness per given area and given sampling effort [1,2]. In plants, diversity assessments are usually plot-based and all species up to accessible heights are sampled (e.g. [3,4]). However, this approach may lead to underestimating the diversity of species not occurring on the ground (i.e. epiphytes). Furthermore, if the proportion of those underestimated species differs among habitat types or varies systematically with some environmental drivers, plot-based diversity assessments will be biased and may result in misleading conservation recommendations. 

Bryophytes and lichens are two very diverse groups of cryptogams, which occur on a wide range of substrates, inhabit essentially all terrestrial and some aquatic habitats and many of them live as epiphytes [5,6]. In forests, the species richness and composition of cryptogam communities varies among geographical regions, forest management types, main tree species, and stand ages [7-10]. On individual trees, the species composition of epiphytic bryophyte and lichen communities changes with tree age, because of changing bark structure as well as quality, and with tree height as abiotic and biotic conditions change from trunk bases to the uppermost twigs [11-15].

Ecological studies, which monitored bryophytes and lichens or compared their species richness in forests among different regions, management types or tree species composition usually sampled all species of a defined area [7,8,15] or only epiphytes on trunks of individual trees [16-18]. However, in these surveys epiphytic species were only sampled up to accessible heights (e.g. 2 m above ground). Thus, epiphytic bryophytes and lichens in tree crowns are rarely investigated, especially for forests in temperate Europe. All previous studies describing the vertical distribution, composition, richness or biomass of epiphytic bryophytes and lichens have been conducted outside continental Europe and mainly investigated single or few fallen or cut trees (e.g. [15,19-35]). 

Only few studies quantified the number of epiphytic bryophyte and lichen species, which are exclusively growing in tree crowns and compared this number to the one of the trunk of the same tree below 2 m [30,35]. However, none of these studies compared the species richness of epiphytic bryophytes and lichens in tree crowns with the total species richness of ground plots, which would have allowed them to assess the number and proportion of overlooked species during regular surveys. Some epiphytes may be recorded on the ground because of epiphytic litter fall and of species growing on fallen branches and twigs, but the true number and proportion of crown specialists remains unknown without surveying tree crowns [36]. Thus, it also remains unclear how many epiphytic bryophyte and lichen species are on average overlooked in regular cryptogam surveys. Furthermore, whether the number or proportion of these overlooked species is consistent among geographical regions, forests types, or stand ages has never been comprehensively analyzed in a comparative study.

Therefore, we sampled the species richness of bryophytes and lichens in European beech (*Fagus sylvatica* L.) and coniferous forests of different stand ages, in three German regions, distinguishing species recorded in a regular survey of all substrates on a plot, epiphytic species occurring only at the base of trees, and epiphytic species occurring only in the crown of trees.

Our main questions were:

1 How many species per tree and per plot are on average overlooked without sampling bryophytes and lichens in tree crowns?
2 Does the number and proportion of overlooked species vary among regions, tree species, and tree age?

## Materials and Methods

### Study system

This study was conducted as part of the Biodiversity Exploratories project ([37]; www.biodiversity-exploratories.de) in three regions of Germany: (1) the UNESCO Biosphere area Schwäbische Alb (Swabian Jura), situated in the low mountain ranges of South-western Germany, (2) the National Park Hainich and its surrounding areas, situated in the hilly lands of Central Germany, and (3) the UNESCO Biosphere Reserve Schorfheide-Chorin, situated in the young glacial lowlands of North-eastern Germany. The three study regions each extend over at least 20 by 30 km. They differ in climate, geology, and topography from each other, while harbouring forest types typical for large parts of temperate Europe (Table 1). 

**Table 1:** Characteristics of the three Biodiversity Exploratories.

	Schwäbische Alb			Hainich-Dün			Schorfheide-Chorin		
Location	SW Germany			Central Germany			NE Germany		
Size	~422 km^2^			~1300 km^2^			~1300 km^2^		
Geology	Calcareous bedrock			Calcareous bedrock			Young glacial landscape		
Altitude a.s.l.	460–860 m			285–550 m			3–140 m		
Annual mean temperature	6.0–7.0 °C			6.5–8.0 °C			8.0–8.5 °C		
Annual mean precipitation	700–1000 mm			500–800 mm			500–600 mm		
Main tree species	European beech		Norway spruce	European beech		Norway spruce	European beech		Scots pine
Number of plots	6		3	9		3	6		3
DBH [m]									
Mean (SD)	0.70 (0.67)		0.30 (0.13)	0.59 (0.27)		0.32 (0.13)	0.58 (0.21)		0.34 (0.16)
Range	0.06–2.01		0.19–0.45	0.03–0.96		0.19–0.45	0.32–0.86		0.16–0.48
Deadwood volume [m^3^]									
Mean (SD)	0.028 (0.019)		0.022 (0.016)	0.033 (0.016)		0.033 (0.008)	0.028 (0.016)		0.020 (0.013)
Range	0.007–0.064		0.012–0.040	0.013–0.067		0.025–0.040	0.010–0.051		0.008–0.035
Ground cover by rocks [%]									
Mean (SD)	0.7 (0.9)		0.0 (0.0)	0.1 (0.2)		0.0 (0.0)	0.2 (0.4)		0.3 (0.3)
Range	0.0–2.5		–	0.0–0.5		–	0.0–1.0		0.0–0.5
Ground cover by bryophytes [%]									
Mean (SD)	1.2 (0.7)		56.0 (46.2)	4.1 (7.9)		36.3 (18.3)	1.3 (1.0)		53.0 (42.9)
Range	0.5–2.0		5.0–95.0	0.5–25.0		22.0–57.0	0.5–3.0		7.0–92.0

Among the forest plots of the Biodiversity Exploratories, we selected the subset of 30 plots (9 plots each in the Schwäbische Alb and Schorfheide-Chorin, and 12 plots in the Hainch-Dün region) dedicated to intensive research. In each region, three plots contain coniferous stands dominated by Norway spruce (*Picea abies* (L.) H.Karst.) in the Schwäbische Alb and Hainich-Dün, and Scots pine (*Pinus sylvestris* L.) in the Schorfheide-Chorin, and six to nine stands dominated by European beech [37]. 

Field work permits were issued by the responsible state environmental offices of Baden-Württemberg, Thüringen, and Brandenburg (according to § 72 BbgNatSchG).

### Bryophyte and lichen sampling

Nomenclature of lichens follows Wirth et al. [38], the one of bryophytes Nebel & Philippi [39-41]. In spring 2008, we first identified bryophyte and lichen species on all 30 plots of 20 m × 20 m, separated by substrate type (bark, rocks, deadwood, and soil), including epiphytic species which were growing on fallen twigs and branches from the tree crown. The sampling of species on bark was restricted to the lower 2 m of trees and shrubs. From this data, we calculated the total number of bryophyte and lichen species occurring in the lower 2 m of the plot. We then selected one tree per plot, which was representative for other trees occurring in the particular plot in terms of tree species identity, height and diameter at breast height (DBH), and recorded epiphytic bryophyte and lichen species which grew in the crown. We used single and double rope climbing techniques, which allowed us to sample approx. 70% of the whole tree crowns, except the outermost branches. We did not limit our sampling time and searched until no more new species were found. We further distinguished species by their substrate affinity into obligate epiphytes, facultative epiphytes, and non-epiphytes according to Wirth et al. [38] for lichens and Nebel & Philippi [39-41] for bryophytes (see Table S1) excluding the ones only identified to genus identity. Differing from Wirth et al. [38] we categorized the lichen *Trapeliopsis flexuosa* as facultative epiphyte instead of a non-epiphyte because the species also occurred as epiphyte in one of our plots. For the same reason we did that also for the bryophyte species *Atrichum undulatum, Eurhynchium praelongum, Plagiomnium undulatum, Polytrichum formosum, Rhytidiadelphus loreus*, and *Thuidium tamariscinum* which were listed as non-epiphytes in Nebel & Philippi [39-41]. 

From this data we calculated the number of species per tree, dividing them into those exclusively growing on the trunk below 2 m, exclusively growing in the crown, and shared species between the trunk and the crown. However, as *Orthotrichum stramineum* was according to Nebel & Philippi [39-41] the only obligate epiphytic bryophyte species in our data set, we did not analyze the three bryophyte groups separately.

### Plot characteristics

We measured the DBH of the investigated trees, which has been shown to be related to tree age [42] and the surface area of the tree crown [43]. Furthermore, we estimated the ground cover by bryophytes and rocks, and measured length and diameter of all deadwood items (≥7 cm of diameter) to estimate deadwood volume assuming cylinder shapes (see Table 1).

### Statistical analysis

We analyzed the number of overlooked bryophyte and lichen species per tree and per plot using GLM models with Poisson errors and corrected for overdispersion, when necessary. Explanatory variables were the three study regions, tree species identity (European beech vs. conifer), and DBH of the sampled tree. Furthermore, we included interactions between explanatory variables. F- and Chi-square tests were used to test the significance of deviance changes associated with factors added sequentially to the model (sequence shown in Table 2). Model simplification did not change the results and we therefore present the full models. Data were analyzed using *R*, Version 2.13.1 [44].

**Table 2:** GLM results for differences in the number of overlooked bryophyte and lichen species among the three regions, European beech vs. **conifer, and tree ages, indicated by the DBH of the investigated tree**.

		Overlooked species per tree					Overlooked species per plot				
		Lichens			Bryophytes		Lichens			Bryophytes	
Source of variation	df	*F*	*p*		*CHI^2^*	*p*	*F*	*p*		*F*	*p*
Region	2	71.68	**0.000**		4.19	0.123	42.73	**0.002**		0.65	0.468
Beech vs. conifer	1	9.71	0.079		19.81	**0.000**	14.03	**0.043**		11.09	**0.000**
DBH	1	20.35	**0.011**		4.95	**0.026**	24.79	**0.007**		4.99	**0.001**
*Interactions*											
Beech vs. conifer × DBH	1	0.78	0.619		1.68	0.194	1.14	0.565		0.00	0.999
Region × beech vs. conifer	2	7.47	0.305		2.28	0.320	4.24	0.540		0.00	0.999
Region × DBH	2	2.40	0.683		2.02	0.365	1.04	0.860		1.00	0.314
Residual Deviance	20	66.54			19.87		71.18			10.70	

Significant differences are indicated by bold p values.

## Results

### Overall and regional species richness

Over all 30 plots, we recorded 99 lichen and 88 bryophyte species. Of the 99 lichen species, 80 were epiphytes (38 obligate epiphytes according to Wirth et al. [38]), 11 were growing on deadwood, 15 on rocks, and 21 on fallen twigs and branches from the tree crown. Interestingly, we found no lichen species growing on soil. Of the 88 bryophyte species, 51 were epiphytes (1 obligate epiphyte according to Nebel & Philippi [39-41]), 38 were growing on deadwood, 29 on rocks, 38 on soil, and 5 on fallen twigs and branches from the tree crown. In the Schwäbische Alb region we recorded 87 lichen and 69 bryophyte species, in Hainich-Dün 25 and 46, and in Schorfheide-Chorin 38 and 30, respectively. Fifty-six lichen and 34 bryophyte species were recorded exclusively in the Schwäbische Alb region, 1 and 6 in Hainich-Dün, as well as 11 and 6 in Schorfheide-Chorin, respectively. Over all three regions, we recorded 15 epiphytic lichen and 3 epiphytic moss species only in tree crowns (for details see Table S1).

### The number and proportion of overlooked epiphytic lichen and bryophytes species

**Figure 1:**
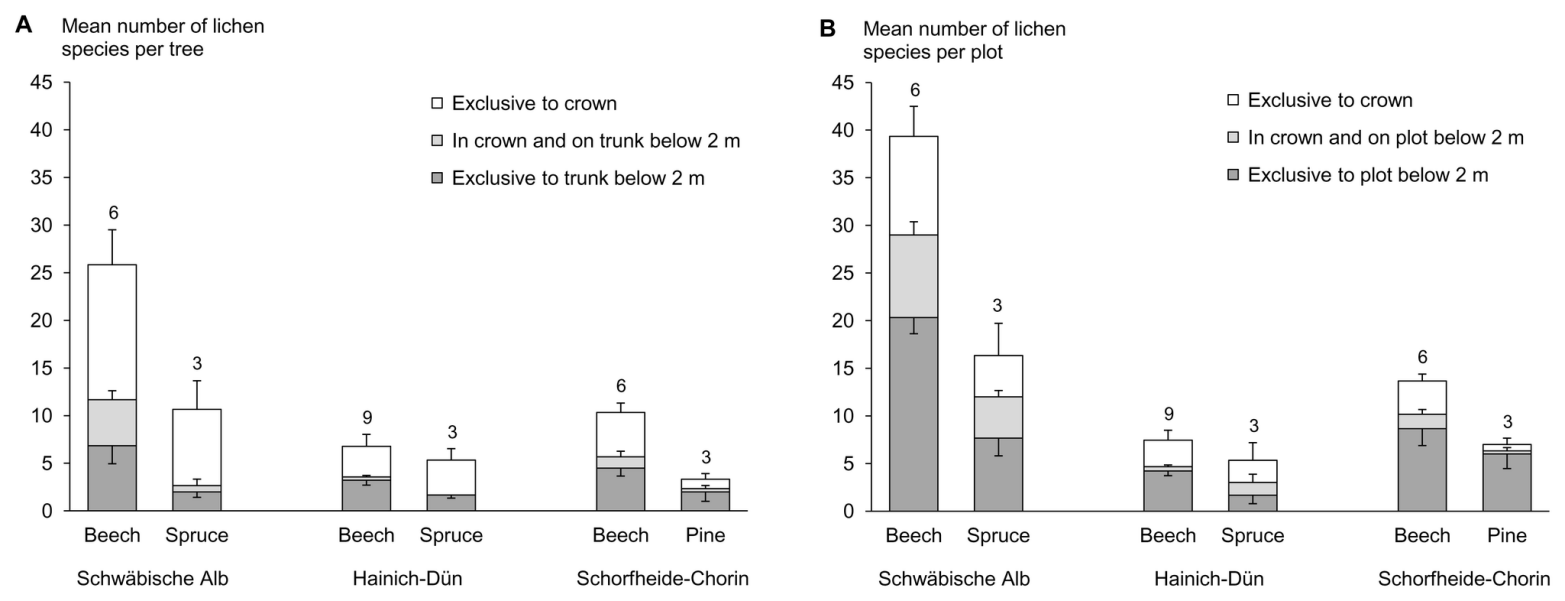
Mean number of lichen species (+SE) growing A) exclusively on the trunk below 2 m, exclusively in the crown, or in both parts of the sampled tree, and B) exclusively on the plot below 2 m, exclusively in the crown of the sampled tree, or in both parts of the plot, separated for the main tree species per plot for all three study regions. Sample size is indicated by the numbers above the bars.

**Figure 2:**
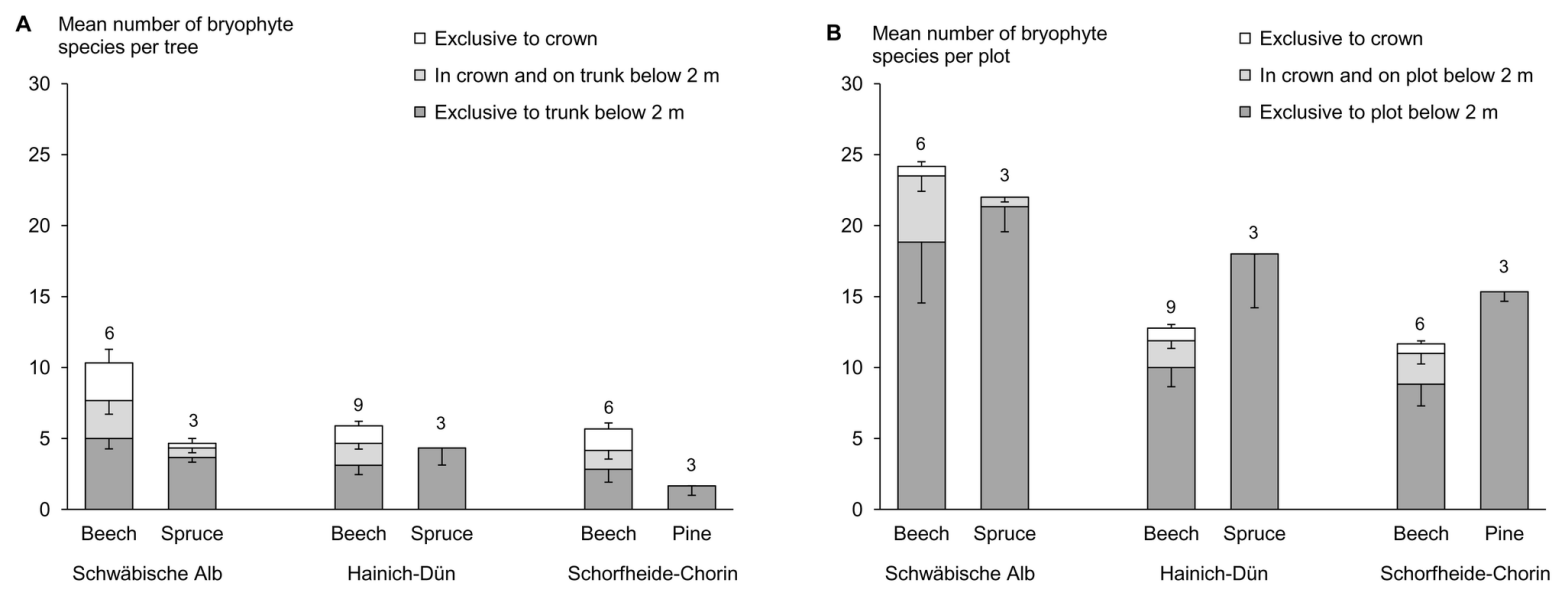
Mean number of bryophyte species (+SE) growing A) exclusively on the trunk below 2 m, exclusively in the crown, or in both parts of the sampled tree, and B) exclusively on the plot below 2 m, exclusively in the crown of the sampled tree, or in both parts of the plot, separated for the main tree species per plot for all three study regions. Sample size is indicated by the numbers above the bars.

At the level of a single tree, a substantial number of epiphytic lichen and bryophyte species were overlooked if the crown had not been sampled. On average, we recorded 11.2 (± 10.3, mean ± standard deviation) epiphytic lichen species per tree of which 6.0 (± 6.4) species occurred exclusively in the crown. For bryophytes, we recorded an average of 6.0 (± 3.8) epiphytic species per tree, of which 1.2 (± 1.5) occurred exclusively in the crown. These data indicate that on average 54% of lichens and 20% of bryophytes were overlooked per tree when the crown was not sampled (Figure 1A, 2A). 

At the level of the plots, by also sampling the crown of one tree per plot we recorded 38% more lichen species and 4% more bryophyte species (Figure 1B, 2B) than by sampling the bark below 2 m and the ground substrates only. Comparing these values with individual tree data indicates that other substrates in a plot harbour a large part of the bryophyte species typical for tree crowns, whereas this was not the case for lichens. Classifying bryophyte and lichen species by their substrate affinity into obligate epiphytes, facultative epiphytes, and non-epiphytes did not qualitatively change the results (Figure S1). These findings indicate that the number of overlooked species per plot is hardly driven by obligate epiphytic species.

### Differences in the number and proportion of overlooked epiphytic lichen and bryophyte species among regions and forest stands

For lichens, the number and proportion of overlooked epiphytic species varied strongly among regions: At the level of trees, on average in the Schwäbische Alb 12.1 (± 2.7, mean ± standard error; 58% of all species), in Hainich-Dün 3.3 (± 1.0; 52%), and in Schorfheide-Chorin 3.4 (± 0.9; 43%) epiphytic lichen species were overlooked if the crown had not been sampled (Figure 1A). At the level of plots, on average in the Schwäbische Alb 8.3 (± 2.5; 26%), in Hainich-Dün 2.7 (± 0.9; 39%), and in Schorfheide-Chorin 2.6 (± 0.7; 22%) epiphytic lichen species were overlooked if the crown of one tree had not been sampled (Figure 1B). Furthermore, on average the number of overlooked epiphytic lichen species per plot was higher in European beech (5.1 ± 1.2 species; 28%) than in coniferous stands (2.4 ± 1.3 species; 26%; Figure 1B; Table 2). 

Finally, the number of overlooked epiphytic lichen species increased by approximately 1 species with an increase of 0.1 m in DBH of the sampled tree, both at the level of trees and plots. These findings indicate that the extent to which species richness was underestimated by ignoring tree crowns varied as a function of local conditions and stand characteristics, and thereby affects biodiversity assessments differently.

For bryophytes the number and proportion of overlooked epiphytic species per tree and per plot did not vary among regions. On average, the number of overlooked epiphytic bryophyte species per tree was higher on European beech (1.7 ± 0.3 species; 24%) than on coniferous trees (0.1 ± 0.1 species; 3%) if the crown had not been sampled (Figure 2A). Furthermore, on average the number of overlooked epiphytic bryophyte species per plot was higher in European beech (0.8 ± 0.2 species; 5%) than in coniferous stands (0.0 ± 0.0 species; 0%) if the crown of one tree had not been sampled (Figure 2B, Table 2). The number of overlooked epiphytic bryophyte species increased by 0.13 species per plot with an increase of 0.1 m in DBH of the sampled tree. These results indicate that bryophyte diversity is underestimated by ignoring tree crowns, especially in beech forests. 

## Discussion

### Overlooked epiphytic bryophyte and lichen species per tree and plot

We found high numbers of species exclusively growing in the canopy of the sampled tree, which are overlooked when only the first 2 meters of the tree trunk are sampled. This number still remained high, at least for lichen species, when all other substrates as well as epiphyte litter fall in a plot were sampled. At the level of trees, lichen [30,35] or bryophyte species [30] exclusive to the crown have rarely been addressed for European forests and not at all for temperate European ones. Among the few studies, Marmor et al. [35] recorded lichens on 15 Norway spruces and 15 Scots pines in southern Estonia and concluded that approximately two third of the trees’ lichen species are growing exclusively in the canopy. Fritz [30] sampled species of conservation concern on European beeches of three age classes in Sweden and found both bryophyte and lichen species which were restricted to the crown of trees. However, across all investigated trees, he found all but one lichen species of conservation concern growing also below 2 m and concluded that the regular surveying method is applicable and efficient for a high number of stems if the aim of a study is to record species of conservation concern within a stand. 

However, none of the previous studies related their findings of differences in bryophyte and lichen species between trunks and crowns of individual trees to the level of a plot with a defined area, although this is the most common method in vegetation surveys with the aim to compare the species richness among different forest types. Thus, it has not been tested how many species on average are overlooked during regular plot-based surveys. We showed that, even after sampling the lower bark of all other trees of the plot, as suggested by Fritz [30] to reduce the number of underestimated species, and all other substrates and epiphyte litter fall, the number of overlooked epiphytic species still remained high, at least for lichen species. For bryophytes, in contrast, sampling of epiphyte litter fall and other substrates seems to reduce the number of overlooked species per plot to a negligible overall value.

### Variation in overlooked epiphytic bryophyte and lichen species among regions and forest stands

In a larger study from the same regions which were investigated here, Boch et al. [8] sampled 631 forest plots with regular sampling methods and reported regional differences in lichen species richness, which were most likely related to the former intensity of atmospheric pollutants. In the current study, the number of overlooked lichen species per plot also differed among regions, whereas this was not the case for bryophytes. This is clearly calling for caution when attempting to generalize results among regions and taxa for proposing conservation recommendations. 

With regard to the overall bryophyte species richness on a plot up to 2 m height, Humphrey et al. [45], who compared deciduous stands and conifer plantations in Britain and found almost exclusively species growing on deadwood, observed no differences in bryophyte richness between deciduous stands and conifer plantations. This is in contrast to our findings of higher bryophyte species richness in coniferous than European beech stands, which was mainly because of generally higher numbers of bryophyte species growing on soils of coniferous stands. In coniferous stands, soil-dwelling bryophytes profit from higher light availability on the forest floor due to less closed canopy cover [46] and reduced litter fall compared with generally unfavorable conditions in European beech forests [47]. However, for lichens we found the opposite of higher overall numbers in European beech than in conifer stands because of higher numbers of epiphytic species. Similarly, Marmor et al. [35] and Humphrey et al. [45] reported differences in the total number and composition of lichen species among tree species. One explanation might be that native tree species provide a more suitable habitat for epiphytic species than non-native ones. In our case, European beech or mixed beech forests would most likely form the potential natural vegetation of all our investigated plots and all coniferous stands are plantations [48]. Furthermore, all investigated coniferous plantations resulted from clear cuts which are poor in species depending on stand continuity [8]. 

With regard to the overlooked epiphytic bryophyte and lichen species on the level of a plot, their number was generally lower in coniferous than European beech stands. This pattern was more pronounced for lichen than for bryophyte species, reflecting generally high numbers of crown specific lichen species. In contrast, crown-specific bryophyte species were almost completely lacking on coniferous trees. One explanation could be that the number of microhabitats for specialized bryophytes, such as rot holes and pronounced bark textures [49] is lower in the crowns of coniferous trees than of European beeches. In addition, microclimatic variables such as light and humidity conditions might likely differ among tree species. However, coniferous trees generally appear to provide less favorable conditions for epiphytic bryophytes than deciduous ones (e.g. [11,50,51]).

In general, the number of overlooked bryophytes and lichens per tree and plot increased with increasing tree age, indicated by the DBH of the investigated tree. This is most likely because older trees provide a range of microhabitats, which make them better suitable for more specialized bryophyte and lichen species (e.g. [30,49,52,53]). 

In conclusion, the number of overlooked bryophyte and lichen species differs among regions and forest types, indicating a bias of comparative studies which might have led to misleading conservation recommendations of plot-based diversity assessments. Even if this bias was smaller for bryophytes than for lichens, with relatively low numbers of overlooked species per plot, it was still significant.

### Recommendations for diversity assessments

Our study emphasizes the importance of tree crown sampling for complete plot-based diversity assessments. As the number of tree crown specialists differs among regions and forest types, it appears indispensable to correct for such differences before comparing their species richness. Thus, further studies are needed developing specific correction factors, which would allow an objective comparison of lichen and bryophyte species richness among forest types and regions, leading to unbiased conservation recommendations. Up to now it remains unclear, how many tree crowns per region, forest type, and plot have to be investigated to capture all canopy specialists and calculate a representative correction factor. Furthermore, it is open how increasing tree diversity is affecting the sampling bias. We are aware that time-consuming methods, such as tree climbing, are not always applicable in regular surveys, in particular, when high numbers of plots need to be sampled. Instead of climbing, bryophytes and lichens can also be sampled from the crowns of recently fallen or cut trees, which represent the majority of the stand dominating trees in terms of tree species identity and tree age. 

## Supporting Information

Figure S1 **Mean number of lichen species (+SE) separated by substrate affinity (according to Wirth et al. [38]) growing A) exclusively on the trunk below 2 m, exclusively in the crown, or in both parts of the sampled tree, and B) exclusively on the plot below 2 m, exclusively in the crown of the sampled tree, or in both parts of the plot, separated for the main tree species per plot for all three study regions.** Sample size is indicated by the numbers above the bars.
(TIF)

Table S1 **Presence of lichen and bryophyte species separated by substrate affinity (according to Wirth et al. [38] for lichens and Nebel & Philippi [39-41] for bryophytes) and the substrate on which they occurred i) in the crown and ii) on the trunk of the sampled tree, and iii) on other substrates in the plot excluding the sampled tree.** Species marked with an asterisk were only found in tree crowns.
(XLSX)

## References

[1] Nimis PL, Scheidegger C, Wolseley PA (2002) Monitoring with Lichens – monitoring lichens. IV. Earth and Environmental Sciences Vol. 7. Dordrecht: Kluwer Academic Publishers. 408 p.

[2] Millennium Ecosystem Assessment (2005) Ecosystems and Human Well-being: Biodiversity Synthesis. Washington, DC: World Resources Institute. 137 p.

[3] Magurran AE (2004) Measuring biological diversity. Oxford: Wiley-Blackwell. 264 p.

[4] Boch S, Prati D, Müller J, Socher S, Baumbach H et al. (2013) High plant species richness indicates management-related disturbances rather than the conservation status of forests. Basic Appl Ecol 14: 496–505. doi:10.1016/j.baae.2013.06.001.

[5] Porley R, Hodgetts N (2005) Mosses & Liverworts. London: HarperCollins. 480 p.

[6] Lutzoni F, Miadlikowska J (2009) Lichens. Curr Biol 19: R502–R503. doi:10.1016/j.cub.2009.04.034. PubMed: 19602407. 19602407

[7] Paillet Y, Bergès L, Hjältén J, Odor P, Avon C et al. (2010) Biodiversity differences between managed and unmanaged forests: meta-analysis of species richness in Europe. Conserv Biol 24: 101–112. doi:10.1111/j.1523-1739.2009.01399.x. PubMed: 20121845. 20121845

[8] Boch S, Prati D, Hessenmöller D, Schulze ED, Fischer M (2013) Richness of lichen species, especially of threatened ones, is promoted by management methods furthering stand continuity. PLOS ONE 8(1): e55461. doi:10.1371/journal.pone.0055461. PubMed: 23383196. 23383196 PMC3558497

[9] Nascimbene J,·Thor G, Nimis PL (2013) Effects of forest management on epiphytic lichens in temperate deciduous forests of Europe – A review. Forest Ecol Manage 298: 27–38. doi:10.1016/j.foreco.2013.03.008.

[10] Svensson M, Dahlberg A, Ranius T, Thor G (2013) Occurrence Patterns of Lichens on Stumps in Young Managed Forests. PLOS ONE 8(4): e62825. doi:10.1371/journal.pone.0062825. PubMed: 23638150. 23638150 PMC3634766

[11] Barkman JJ (1958) Phytosociology and ecology of cryptogamic epiphytes. Assen: van Gorcum. 628 p.

[12] Cornelissen JHC, Ter Steege H (1989) Distribution and ecology of epiphytic bryophytes and lichens in dry evergreen forest of Guyana. J Trop Ecol 5: 131–150. doi:10.1017/S0266467400003400.

[13] Mazimpaka V, Lara F (1995) Corticolous bryophytes of *Quercus* *pyrenaica* forests from Gredos Mountains (Spain): vertical distribution and affinity for epiphytic habitats. Nova Hedwigia 61: 431–446.

[14] Sillett SC, Antoine ME (2004) Lichens and bryophytes in forest canopies. In: Lowman MD Rinker HB Forest Canopies, 2nd ed. New York: Elsevier Academic Press pp. 151–174.

[15] Fritz Ö, Gustafsson L, Larsson K (2008) Does forest continuity matter in conservation? A study of epiphytic lichens and bryophytes in beech forests of southern Sweden. Biol Conserv 141: 655–668. doi:10.1016/j.biocon.2007.12.006.

[16] Friedel A, von Oheimb G, Dengler J, Haerdtle W (2006) Species diversity and species composition of epiphytic bryophytes and lichens – a comparison of managed and unmanaged beech forests in NE Germany. Feddes Repert 117: 172–185. doi:10.1002/fedr.200511084.

[17] Moning C, Werth S, Dziock F, Bässler C, Bradtka J et al. (2009) Lichen diversity in temperate montane forests is influenced by forest structure more than climate. Basic Appl Ecol 258: 745–751.

[18] Jönsson MT, Thor G (2012) Estimating Coextinction Risks from Epidemic Tree Death: Affiliate Lichen Communities among Diseased Host Tree Populations of *Fraxinus* *excelsior* . PLOS ONE 7(9): e45701. doi:10.1371/journal.pone.0045701. PubMed: 23049840. 23049840 PMC3458109

[19] Harris GP (1971) The Ecology of Corticolous Lichens: 1. The Zonation on Oak and Birch in South Devon. J Ecol 59: 431–439. doi:10.2307/2258323.

[20] Eversman S, Johnson C, Gustafson D (1987) Vertical distribution of epiphytic lichens on three tree species in Yellowstone National Park. Bryologist 90: 212–216. doi:10.2307/3242928.

[21] Wolf JHD (1993) Diversity patterns and biomass of epiphytic bryophytes and lichens along an altitudinal gradient in the northern Andes. Ann Mo Bot Gard 80: 928–960. doi:10.2307/2399938.

[22] Jarman SJ, Kantvilas G (1995) Epiphytes on an old Huon pine tree (*Lagarostrobos* *franklinii*) in Tasmanian rainforest. NZJ Bot 33: 65–78. doi:10.1080/0028825X.1995.10412944.

[23] Sillett SC, Gradstein SR, Griffin D (1995) Bryophyte diversity of ficus tree crowns from cloud forest and pasture in Costa Rica. Bryologist 98: 251–260. doi:10.2307/3243312.

[24] Aptroot A (1997) Lichen biodiversity in Papua New Guinea, with the report of 173 species on one tree. Bibl Lichenol 68: 203–213.

[25] McCune B, Amsberry KA, Camacho FJ, Clery S, Cole C et al. (1997) Vertical Profile of Epiphytes in a Pacific Northwest Old-growth Forest. Northwest Sci 71: 145–152.

[26] Milne J, Louwhoff S (1999) Vertical distribution of bryophytes and lichens on a Myrtle Beech, *Nothofagus* *cunninghamii* (Hook.). OerstHikobia 13: 23–30.

[27] Sillett SC, Rambo TR (2000) Vertical distribution of dominant epiphytes in Douglas-fir forests of the Central Oregon Cascades. Northwest Sci 74: 44–49.

[28] Holz I, Gradstein SR, Heinrichs J, Kappelle M (2002) Bryophyte diversity, microhabitat differentiation, and distribution of life forms in Costa Rican upper montane *Quercus* forest. Bryologist 105: 334–348. Available online at: doi:10.1639/0007-2745(2002)105[0334:BDMDAD]2.0.CO;2

[29] Johansson T, Knutsson T, Lundkvist H (2003) Epiphytic lichens on old oaks—what hides on 2–14 meters height? Graph Scr 14: 49–54.

[30] Fritz Ö (2009) Vertical distribution of epiphytic bryophytes and lichens emphasizes the importance of old beeches in conservation. Biodivers Conserv 18: 289–304. doi:10.1007/s10531-008-9483-4.

[31] Diaz IA, Sieving KE, Pena-Foxon ME, Larrain J, Armesto JJ (2010) Epiphyte diversity and biomass loads of canopy emergent trees in Chilean temperate rain forests: A neglected functional component. Forest Ecol Manage 259: 1490–1501. doi:10.1016/j.foreco.2010.01.025.

[32] Normann F, Weigelt P, Gehrig-Downie C, Gradstein SR, Sipman HJM et al. (2010) Diversity and vertical distribution of epiphytic macrolichens in lowland rain forest and lowland cloud forest of French Guiana. Ecol Indic 10: 1111–1118. doi:10.1016/j.ecolind.2010.03.008.

[33] Sporn SG, Bos MM, Kessler M, Gradstein SR (2010) Vertical distribution of epiphytic bryophytes in an Indonesian rainforest. Biodivers Conserv 19: 745–760. doi:10.1007/s10531-009-9731-2.

[34] Romanski J, Pharo EJ, Kirkpatrick JB (2011) Epiphytic bryophytes and habitat variation in montane rainforest, Peru. Bryologist 114: 720–731. doi:10.1639/0007-2745-114.4.720.

[35] Marmor L, Tõrra T, Saag L, Leppik E, Randlane T (2013) Lichens on *Picea* *abies* and *Pinus* *sylvestris* – from tree bottom to the top. Lichenologist 45: 51–63. doi:10.1017/S0024282912000564.

[36] Will-Wolf S, Esseen PA, Neitlich P (2002) Monitoring biodiversity and ecosystem function: forests. In: Nimis PL Scheidegger C Wolseley PA Monitoring with lichens – monitoring lichens, IV. Earth and Environmental Sciences Vol. 7. Dordrecht: Kluwer Academic Publishers pp. 203–222.

[37] Fischer M, Bossdorf O, Gockel S, Hänsel F, Hemp A et al. (2010) Implementing large-scale and long-term functional biodiversity research: The Biodiversity Exploratories. Basic Appl Ecol 11: 473–485. doi:10.1016/j.baae.2010.07.009.

[38] Wirth V, Hauck M, Schultz M, editors (2013) Die Flechten Deutschlands. Stuttgart: Ulmer.

[39] Nebel M, Philippi G, editors (2000) Die Moose Baden-Württembergs. Band 1 Stuttgart: Ulmer.

[40] Nebel M, Philippi G, editors (2001) Die Moose Baden-Württembergs. Band 2 Stuttgart: Ulmer.

[41] Nebel M, Philippi G, editors (2005) Die Moose Baden-Württembergs. Band 3 Stuttgart: Ulmer.

[42] Nascimbene J,·Marini L,·Motta R, Nimis PL (2009) Influence of tree age, tree size and crown structure on lichen communities in mature Alpine spruce forests. Biodivers Conserv 18:1509–1522.

[43] Rédei K, Veperdi I (2001) Study of the relationships between crown and volume production of black locust trees (*Robinia* *pseudoacacia* L.). Lesn. Čas. Forestry Journal 47: 135–142.

[44] R Development Core Team (2011) R: a language and environment for statistical computing. R Foundation for Statistical Computing, Vienna.

[45] Humphrey JW, Davey S, Peace AJ, Ferris R, Harding K (2002) Lichens and bryophyte communities of planted and semi-natural forests in Britain: the influence of site type, stand structure and dead wood. Biol Conserv 107: 165–180. doi:10.1016/S0006-3207(02)00057-5.

[46] Tinya F, Márialigeti S, Kiraly I, Nemeth B, Ódor P (2009) The effect of light conditions on herbs, bryophytes and seedlings of temperate mixed forests in Őrség, Western Hungary. Plant Ecol 204: 69–81. doi:10.1007/s11258-008-9566-z.

[47] Márialigeti S, Németh B, Tinya F, Ódor P (2009) The effects of stand structure on ground-floor bryophyte assemblages in temperate mixed forests. Biodiversity Conserv 18: 2223–2241. doi:10.1007/s10531-009-9586-6.

[48] Bohn U, Neuhäusl R (2000) Karte der natürlichen Vegetation Europas – Karten. BfN, Bonn.

[49] Fritz Ö, Niklasson M, Churski M (2008) Tree age is a key factor for the conservation of epiphytic lichens and bryophytes in beech forests. Appl Veg Sci 12: 93–106.

[50] Fengel D, Wegener G (1984) Wood: chemistry, ultrastructure, reactions. Berlin: Walter de Gruyter.

[51] Király I, Ódor P (2010) The effect of stand structure and tree species composition on epiphytic bryophytes in mixed deciduous–coniferous forests of Western Hungary. Biol Conserv 143: 2063–2069. doi:10.1016/j.biocon.2010.05.014.

[52] Ranius T, Johansson P, Berg N, Niklasson M (2008) The influence of forest age and microhabitat quality on the occurrence of crustose lichens associated with old oaks. J Veg Sci 19: 653–662. doi:10.3170/2008-8-18433.

[53] Fritz Ö, Heilmann-Clausen J (2010) Rot holes create key microhabitats for epiphytic lichens and bryophytes on beech (*Fagus* *sylvatica*). Biol Conserv 143: 1008–1016. doi:10.1016/j.biocon.2010.01.016.

