# Supplementary figures and images for: Up in the Tree – The Overlooked Richness of Bryophytes and Lichens in Tree Crowns
Source: PLoS One. 2013 Dec 17;8(12):e84913. doi: 10.1371/journal.pone.0084913 (PMC3866205; doi:10.1371/journal.pone.0084913)

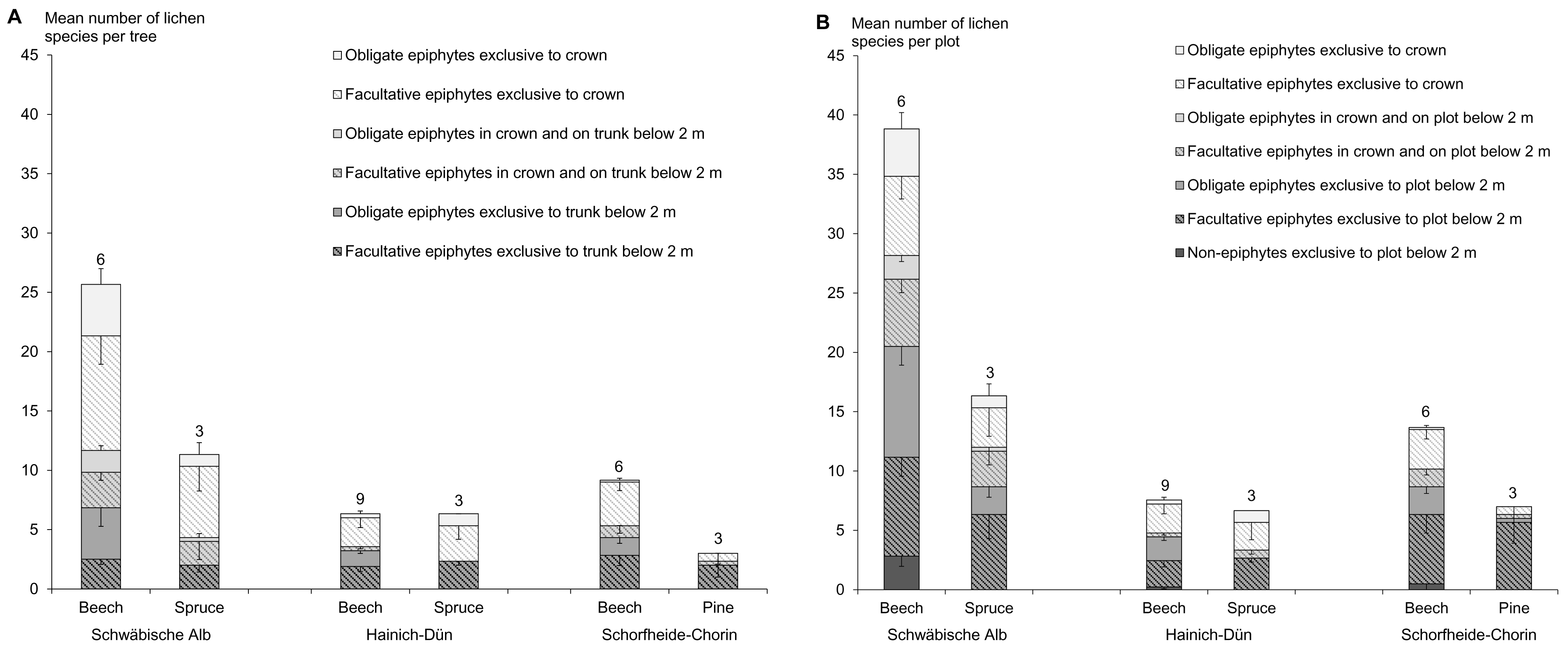

Supplement: Figure S1 — Mean number of lichen species (+SE) separated by substrate affinity (according to Wirth et al. [38]) growing A) exclusively on the trunk below 2 m, exclusively in the crown, or in both parts of the sampled tree, and B) exclusively on the plot below 2 m, exclusively in the crown of the sampled tree, or in both parts of the plot, separated for the main tree species per plot for all three study regions. Sample size is indicated by the numbers above the bars. (TIF) [file pone.0084913.s001.tif]
